# Roles of 21 Genera of Human Gut Microbiota in Barrett’s Esophagus Risk: A Mendelian Randomization Study

**DOI:** 10.3389/fgene.2022.894900

**Published:** 2022-06-09

**Authors:** Zhao Yang, Rong Yu, Wei Deng, Weihu Wang

**Affiliations:** ^1^ School of Public Health, Li Ka Shing Faculty of Medicine, The University of Hong Kong, Hong Kong, China; ^2^ Key Laboratory of Carcinogenesis and Translational Research (Ministry of Education/Beijing), Department of Radiation Oncology, Peking University Cancer Hospital and Institute, Beijing, China

**Keywords:** Barrett’s esophagus, causal estimates, gut microbiota, Mendelian randomization, MiBioGen

## Abstract

**Background:** Lack of definitive evidence supports the putative hypothesis that gut microbiota dysbiosis is associated with Barrett’s esophagus (BE). We conducted a two-sample Mendelian randomization study to assess the associations of 21 genera of human gut microbiota with BE.

**Methods:** We identified independent genetic instruments for 21 genera of gut microbiota (including nine dominant genera, four core genera among individuals of European ancestry, and eight esophagus-specific genera of gut microbiota) from MiBioGen (up to 18,340 participants). We applied them to summary statistics from the largest publicly available genome-wide association study on BE (9,680 cases and 31,211 controls). We obtained the causal estimates of genetically predicted higher genera of gut microbiota and BE using the inverse variance weighting method. Sensitivity analyses included weighted median, MR-Egger, MR-RAPS, and MR-PRESSO.

**Results:** We found that genetically predicted higher *Actinomyces* (OR: 0.76 per unit increase in log odds of having BE, 95% CI: 0.70–0.83) and higher *Ruminiclostridium* (OR: 0.75, 95% CI: 0.63–0.90) were significantly associated with a lower risk of BE. No associations of other genera of gut microbiota with BE were noted, apart from suggestive associations of higher *Alistipes* (OR: 0.77; 95% CI: 0.61–0.99), higher *Eubacterium* (OR: 0.89; 95% CI: 0.80–0.99), and higher *Veillonella* (OR: 0.76; 95% CI: 0.56–1.02) with a lower risk of BE, and higher *Faecalibacterium* (OR: 1.15; 95% CI: 0.99–1.33) with a higher risk of BE.

**Conclusion:** This study suggests that higher *Actinomyces* and higher *Ruminiclostridium* might protect against BE.

## Introduction

The International Agency for Research on Cancer reported that around 15%–20% of cancer cases were attributable to microbial, particularly commensal microbiota (IARC Working Group on the Evaluation of Carcinogenic Risks to Humans, 2012). The human gastrointestinal (GI) tract, harboring up to ten thousand billion individual non–site-specific bacteria and three million bacterial genes, has recently become the focus of great interest as target interventions to combat Barrett’s esophagus (BE) and alleviate the side effects of therapeutics on GI cancer (Bhatt et al., 2017; Gillespie et al., 2021). Generally, dysbiosis changing the composition and function of gut microbiota appears to manipulate the host immune system and produce metabolites, which have been implicated in the development of BE and esophageal adenocarcinoma (EAC) (Bhatt et al., 2017; Levy et al., 2017). Several possible underlying mechanisms support such a hypothesis, for example, the loss of tolerance by the host immune system, modulating inflammation, inducing DNA damage, producing metabolites involved in tumor initiation and progression, and maladaptation of the host’s gut environment (Turnbaugh et al., 2007; Tozun and Vardareli, 2016; Bhatt et al., 2017; Levy et al., 2017). However, randomized controlled trials investigating the association of gut microbiota with BE remain scarce.

Observationally, several gut microbiomes have been reported to associate with BE. For example, a small-scale case-control study (ten BE cases and ten controls) showed that a decreased *Streptococcus* and increased *Prevotella*, *Veillonella*, and *Leptotrichia* were associated with a high BE risk (Lopetuso et al., 2020). The consistent result of a decreased *Streptococcus* on BE risk was also observed by another small-size study (Yang et al., 2009). In addition, increased *Proteobacteria*, *Enterobacteriaceae*, and *Akkermansia* were reported to associate with high-grade dysplasia risk (Snider et al., 2019). However, these results are usually difficult to interpret and cannot distinguish effects of changes in genera of gut microbiota from effects of confounding (e.g., diet and obesity), despite differences in sample collections, analysis methods, and study populations.

Mendelian randomization (MR) studies provide a valuable framework to investigate their roles in BE as it resists confounding by utilizing genetic variants randomly allocated at conception to proxy the exposure of interest (Smith and Ebrahim, 2003; Lawlor et al., 2008). More importantly, no MR study has been conducted to assess the possible gut microbiota–BE associations. To this end, we conducted a two-sample MR study using summary statistics from the largest publicly available genome-wide association study (GWAS) of gut microbiota and BE (An et al., 2019; Kurilshikov et al., 2021) in this study. We conducted a narrowed systematic review (Pei et al., 2004; Zilberstein et al., 2007; Yang et al., 2009; Blackett et al., 2013; Elliott et al., 2017; Dong et al., 2018; Hughes et al., 2020; Peter et al., 2020) and considered thirteen genera of human gut microbiota (i.e., *Alistipes*, *Bacteroides*, *Blautia*, *Dorea*, *Faecalibacterium*, *Lachnoclostridium*, *Roseburia*, *Ruminococcus*, *Subdoligranulum*, *Ruminiclostridium*, *Fusicatenibacter*, *Butyricicoccus*, and *Eubacterium*, in which *Ruminiclostridium*, *Fusicatenibacter*, *Butyricicoccus*, and *Eubacterium* are the core genera in the European descent) and eight esophagus-specific genera of gut microbiota (i.e., *Actinomyces*, *Bifidobacterium*, *Haemophilus*, *Peptococcus*, *Lactobacillus*, Prevotella, *Streptococcus*, and *Veillonella*) as shown in Table 1.

**TABLE 1:** Details for 21 genera of human gut microbiota included in this study.

Genus	Id in MiBioGen	N/N of non-zeros	Abundance (per 10 K)	Type^a^	# Candidate SNPs	Ranges of F statistics	R^2,^ ^b^ (%)
Gut (multi-ancestry) Hughes et al. (2020)	—	—	—	—	—	—	—
*Alistipes*	Id.968	18,340/17,571	301.36	mbQTL	15	18.04–23.37	0.94
*Bacteroides*	Id.918	18,302/18,302	1865.24	mbQTL	12	20.04–16.18	0.52
*Blautia*	Id.1992	18,340/18,276	334.91	mbQTL	13	18.59–25.28	0.82
*Dorea*	Id.1997	18,340/17,610	79.98	mbQTL	12	19.50–25.11	1.10
*Faecalibacterium*	Id.2057	18,340/18,087	652.09	mbQTL	13	18.30–33.45	0.66
*Lachnoclostridium*	Id.11308	18,340/17,922	70.59	mbQTL	15	17.05–16.96	0.49
*Roseburia*	Id.2012	18,340/17,854	128.65	mbQTL	18	18.67 to 26.01	0.90
*Ruminococcus*	Id.11373	18,340/16,607	119.15	mbQTL/mbBTL	14	19.03–33.84	0.58
*Subdoligranulum*	Id.2070	18,340/17,591	256.78	mbQTL	14	19.49–26.36	1.15
Gut (European ancestry) Hughes et al. (2020)	—	—	—	—	—	—	—
*Butyricicoccus*	Id.2055	18,340/17,136	33.70	mbQTL	9	19.59–24.47	0.80
*Fusicatenibacter*	Id.11305	18,340/17,384	99.64	mbQTL	20	19.26–24.24	1.11
*Ruminiclostridium*	Id.11355	18,340/17,389	37.58	mbQTL	15	18.53–24.12	0.97
*Eubacterium*	Id.11340	18,340/7,739	76.70	mbQTL/mbBTL	19	19.17–24.98	2.95
Esophagus (multi-ancestry)	—	—	—	—	—	—	—
*Actinomyces* Yang et al. (2009), Blackett et al. (2013)	Id.423	16,762/7,468	3.33	mbQTL	8	19.51–22.24	0.82
*Bifidobacterium* Blackett et al. (2013)	Id.436	18,340/17,571	224.06	mbQTL/mbBTL	18	19.70–88.43	0.74
*Haemophilus* Yang et al. (2009)	Id.3698	18,430/9,119	29.22	mbQTL/mbBTL	14	19.25–29.34	1.74
*Lactobacillus* Zilberstein et al. (2007), Peter et al. (2020)	Id.1837	18,340/6,958	22.57	mbQTL/mbBTL	12	20.28–23.49	1.36
*Peptococcus* Zilberstein et al. (2007)	Id.2037	17,243/5,657	9.65	mbQTL/mbBTL	17	19.51–32.26	2.36
*Prevotella* Pei et al. (2004), Yang et al. (2009), Blackett et al. (2013), Dong et al. (2018), Peter et al. (2020)	id.11183	18,340/10,271	787.49	mbQTL/mbBTL	20	19.35–24.23	2.53
*Streptococcus* Yang et al. (2009), Blackett et al. (2013), Dong et al. (2018), Peter et al. (2020)	Id.1853	18,340/16,387	56.80	mbQTL/mbBTL	18	19.32–36.57	0.40
*Veillonella* Pei et al. (2004), Yang et al. (2009), Elliott et al. (2017), Dong et al. (2018)	Id.2198	18,340/9,291	21.34	mbQTL/mbBTL	11	19.90–23.18	0.71

a Only the taxa with 10%+ of the samples were analyzed as a continuous variable (i.e., mbQTL), whereas taxa present between 10% and 90% of the samples were analyzed as a binary variable (i.e., mbBTL). Specifically, study-specific cutoff for mbQTL analysis was conducted with effective samples being 3,000+ and the presence in at least three cohorts; for mbBTL was conducted among taxa with the mean abundance higher than 1% of in the taxon-positive samples.

b R2: the variance of each gut microbiota genus explained by the selected genetic instruments.

## Methods

### Study Design

This is a two-sample MR study that rests on three key assumptions for inferring causality (Smith and Ebrahim, 2003; Lawlor et al., 2008). First, the genetic instruments strongly predict the exposure of interest (i.e., the relevance assumption). Second, the genetic instruments are independent of confounders of the exposure–outcome association (i.e., the independence assumption). Third, the genetic instruments affect the outcome only *via* the exposure of interest (i.e., the exclusion–restriction assumption).

### Exposure *GWAS*: 21 Genera of Gut Microbiota

We extracted genetic instruments, i.e., single-nucleotide polymorphism (SNP), for 21 genera of gut microbiota from summary statistics in MiBioGen, which is the largest and latest available 16S fecal microbiome data (up to 18,340 participants, including 16,632 adolescents and 1,708 children) on gut microbiota of individuals dominated by of European descent (∼72.3%, 13,266 participants) (Kurilshikov et al., 2021). We selected instruments suggestively [i.e., *p* < 5 × 10^–6^, which is commonly used to highlight “suggestive” genetic variants (Manolio, 2010)] and independently (i.e., r^2^ < 0.01, based on the 1000G European population reference panel) associated with each genus of gut microbiota. The median age of participants in MiBioGen was ∼46.5 years (range: 4–89 years) with ∼44.2% (∼8,073 participants) of males. The genetic effects on genera of gut microbiota were meta-analyzed using either the abundance levels (mbQTL, i.e., samples with zero abundance were truncated) or a binary trait coding presence/absence (mbBTL, i.e., presence versus absence of the bacterial genus), depending on their absolute abundance levels across sub-cohorts. These microbiome GWASs adjusted for age, sex, study-specific covariates, and the top genetic principal components for population stratification. Additional file 1: Supplementary Table S1 shows the list of genetic instruments for genera of gut microbiota included in this study.

### Outcome *GWAS*: BE

We extracted summary statistics of BE from the most recent meta-analyzed BE GWAS (access *via* study accession GCST90000515 (Ong et al., 2021), including 3,513 cases and 14,052 controls in UK Biobank (An et al., 2019); and 6,167 cases and 17,159 controls in a sub-meta BE GWAS (Gharahkhani et al., 2016)) based on the rs number of the identified genetic instruments for 21 genera. Specifically, for UK Biobank, BE and EAC diagnosed with ICD-10 codes (i.e., K22.7 for BE and C15 for EAC) based on self-report and clinical diagnosis were combined as one phenotype mainly because BE is the premalignant precursor of EAC and has a very high genetic correlation with EAC (An et al., 2019). For the sub-meta BE GWAS of all individuals of European descent, patients with BE were identified by the histopathological diagnosis of intestinal metaplasia (Gharahkhani et al., 2016). The meta-analyzed BE GWAS adjusted for age, sex, study-specific covariates, and the first 10 principal components. Additional file 1: Supplementary Table S1 shows the genetic instrument associations with BE.

### Pleiotropic Effects

Given that previous studies (including MR and systematic reviews) showed potential roles of obesity (Thrift et al., 2014), depression (Ong et al., 2021), years of schooling (Ong et al., 2021), and cigarette smoking (Andrici et al., 2013; Cook et al., 2012), we further explored associations of the genetic instruments strongly predicted genera of gut microbiota in respective GWASs with obesity proxied by the waist–hip ratio (GIANT and UK Biobank, *n* = 697,924 participants) (Pulit et al., 2019), cigarette smoking proxied by cigarettes smoked per day (a meta-analyzed GWAS, *n* = 337,334 participants) (Liu et al., 2019), depression (a meta-analyzed GWAS, *n* = 246,363 cases and 561,191 controls) (Howard et al., 2019), and years of schooling (SSGAC, up to 1,131,881 participants) (Lee et al., 2018). Details about study participants included in these GWASs are presented in Additional file 1: Supplementary Table S2. We excluded the instrument associated with any of these phenotypes aforementioned at genome-wide significance (*p* < 5 × 10^−8^) to reduce the possibility of pleiotropy. Here, we did not consider the potential pleiotropic effects of GERD on BE because they shared considerable genetic variants, responding for ∼91% (An et al., 2019). In addition, we cross-checked the associations of the selected instruments with any causes of death using the comprehensively genotype-to-phenotype cross-reference PhenoScanner at *p* < 5 × 10^−8^ to reduce other sources of pleiotropy and the risk of selection bias (Kamat et al., 2019; Schooling et al., 2020; Yang et al., 2021). Additional file 1: Supplementary Table S3 shows the excluded instruments and the corresponding pleiotropic effects.

### Statistical Analysis

We assessed the instrument strength using the F statistic, whose value less than 10 indicated a higher likelihood of weak instrument bias (Bowden et al., 2016a). We used the multiplicative random-effects inverse-variance weighted method to estimate the association of genetically predicted higher genus of gut microbiota on BE by pooling the Wald estimator [i.e., the ratio between the SNP-outcome effect and the SNP-exposure effect with its standard error being approximated using the first-order weights (Bowden et al., 2017)] of each SNP. We reported the heterogeneity of the Wald estimator using the Cochrane Q statistics (Bowden et al., 2017), with the potential directional pleiotropy indicated by *p*<0.05 for MR-Egger intercept (Bowden et al., 2015).

We also conducted the sensitivity analysis using a weighted median estimator (Bowden et al., 2016b), MR-Egger (Bowden et al., 2015; Burgess and Thompson, 2017), MR-PRESSO (Verbanck et al., 2018), and MR robust adjusted profile score (MR-RAPS) (Zhao et al., 2020). Specifically, the weighted median method allows for up to 50% of weights from invalid instruments and produces a consistent causal estimate (Bowden et al., 2016b). MR-Egger detects the directional pleiotropy using the *p* value for the intercept and provides causal estimates after adjusting for pleiotropic effects with an additional assumption about the instrument strength independent of the dependent effect (i.e., InSIDE assumption) (Bowden et al., 2015; Burgess and Thompson, 2017). The MR-PRESSO detects the outlying instruments and provides consistent causal estimates after removing possible outliers with an additional assumption of no invalid instruments (Verbanck et al., 2018). The MR-RAPS allows the inclusion of weak instruments and the presence of systematic and idiosyncratic pleiotropy and provides consistent causal estimates using an adjusted profile likelihood estimator (Zhao et al., 2020).

### Power Analysis

We approximated the variance of each genus of gut microbiota explained by the included instruments using well-established methods for continuous and binary exposures (Lee et al., 2012; Yarmolinsky et al., 2018). We assessed the power of our MR analyses using the online calculator mRnd (https://shiny.cnsgenomics.com/mRnd/) (Brion et al., 2013). Additional file 1: Supplementary Table S1 presents the estimated variance of each genus explained by the instruments. Additional file: Supplementary Table S4 shows the power of the estimated gut microbiota–BE associations.

All analyses were performed using R Version 3.6.2 (R Core Team (2019). R: A language and environment for statistical computing (R Foundation for Statistical Computing, Vienna, Austria. https://www.R-project.org/), with the R package “TwoSampleMR” (Hemani et al., 2018). We reported a two-sided *p* value at the Bonferroni-corrected threshold of 0.05/22 = 0.002, and the *p* value between 0.002 and 0.05 was considered suggestive of causation. We adhere to STROBE-MR: Guidelines for strengthening the reporting of observational studies in epidemiological studies using Mendelian randomization for reporting our results (Skrivankova et al., 2021).

### Ethics Approval

This analysis of publicly available data does not require ethical approval.

## Results

Up to 15 genetic instruments for *Alistipes*, 12 instruments for *Bacteroides*, 13 instruments for *Blautia*, 12 instruments for *Dorea*, 13 instruments for *Faecalibacterium*, 15 instruments for *Lachnoclostridium*, 18 instruments for *Roseburia*, 14 instruments for *Ruminococcus*, 14 instruments for *Subdoligranulum*, 9 instruments for *Butyricicoccus*, 20 instruments for *Fusicatenibacter*, 15 instruments for *Ruminiclostridium*, 19 instruments for *Eubacterium*, 8 instruments for *Actinomyces*, 18 instruments for *Bifidobacterium*, 14 instruments for *Haemophilus*, 12 instruments for *Lactobacillus*, 17 instruments for *Peptococcus*, 20 instruments for *Prevotella*, 18 instruments for *Streptococcus*, and 11 instruments for *Veillonella* were used in this study, as shown in Table 1. All instruments had F statistics (ranging from 17.0 to 88.4) greater than 10, implying a less likely weak instrument bias. Two genetic instruments associated with obesity (rs182549) and depression (rs17708276) were identified and excluded from the MR analysis (Additional file 1: Supplementary Table S3).

**FIGURE 1:**
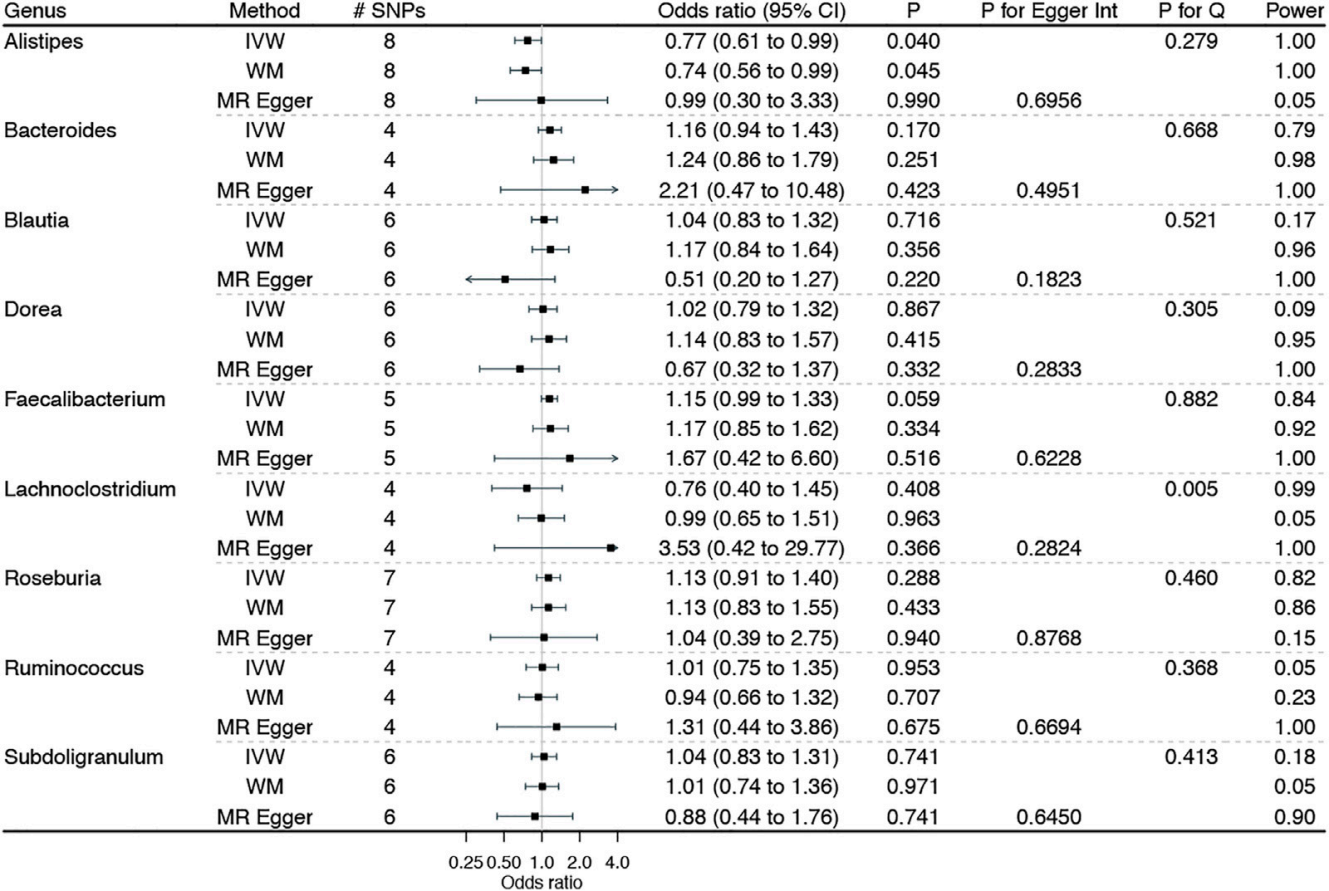
Associations of genetically predicted genera of gut microbiota on Barrett’s esophagus using Mendelian randomization. IVW: inverse variance weighted method with multiplicative random-effects; WM: weighted median estimator.

Figure 1 shows the associations of genetically predicted genera of gut microbiota with BE, with complete results presented in Additional file 1: Supplementary Table S4. No genera of gut microbiota–BE associations were observed after adjusting for multiplicity. However, suggestive associations of genetically predicted higher *Alistipes* with a lower BE risk and higher *Faecalibacterium* with a higher BE risk were noted, with no heterogeneity identified by the Cochran’s Q statistics test. The MR-Egger intercept indicated no horizontal pleiotropy. Furthermore, MR analyses had adequate power (i.e., 80%) to detect a significant association of a genus with BE at α = 0.05 level, given the true association exists. However, the corresponding estimates from MR-Egger regression were not always consistent with the main results, with wide confidence intervals and even reversed causal directions.

**FIGURE 2:**
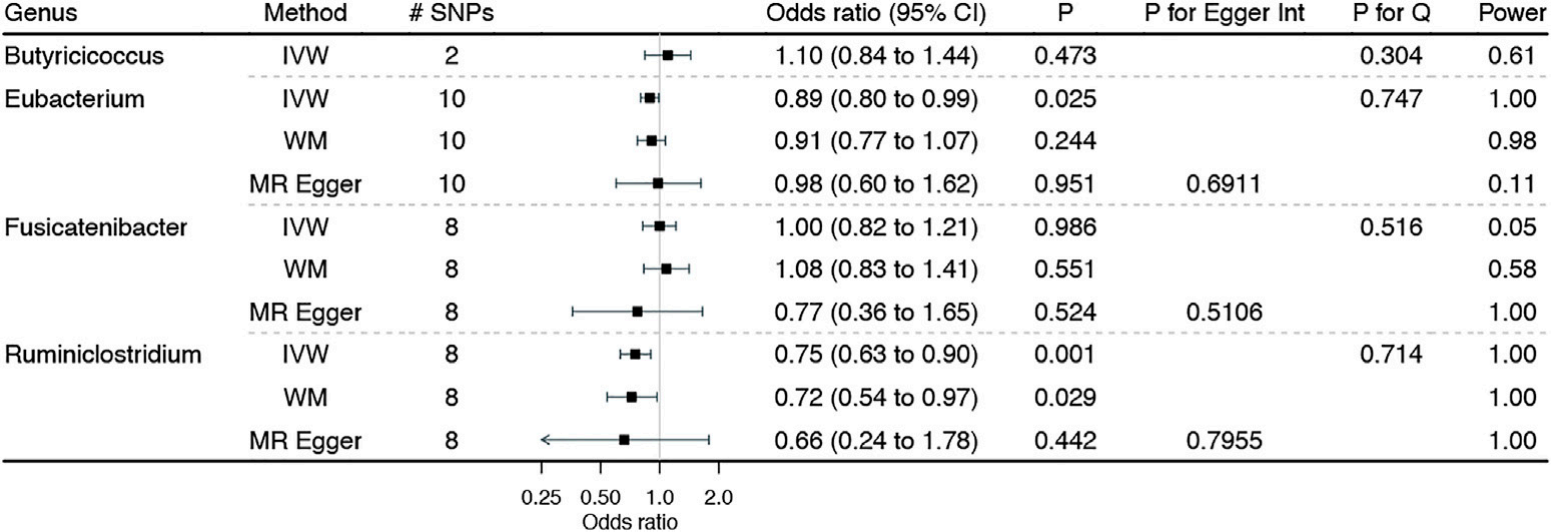
Associations of genetically predicted genera of gut microbiota dominated among individuals of European ancestry on Barrett’s esophagus. IVW: inverse variance weighted method with multiplicative random effects expect for *Butyricicoccus* using the fixed-effect inverse variance weighted method due to the inclusion of two genetic instruments; WM: weighted median estimator.

Figure 2 shows the associations of genetically predicted genera of gut microbiota dominated among individuals from European ancestry with BE, with complete results presented in Additional file 1: Supplementary Table S4. Genetically predicted higher *Ruminiclostridium* was significantly associated with a lower BE risk. The Cochran’s Q statistics test indicated no heterogeneity, and the MR Egger intercept suggested no horizontal pleiotropy. Similarly, higher *Eubacterium* was suggestively associated with a lower BE risk, with no identified heterogeneity and horizontal pleiotropy. Consistent results were also noted from sensitivity analyses, although not reach the Bonferroni-corrected significance.

**FIGURE 3:**
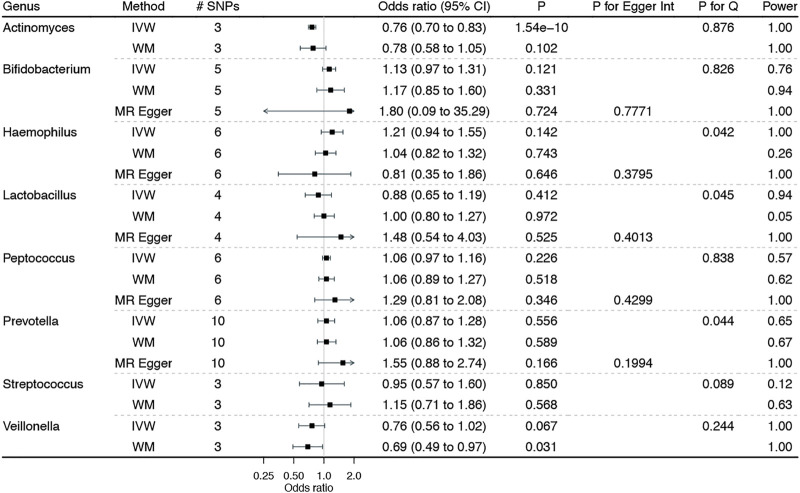
Associations of genetically predicted esophagus-specific genera of gut microbiota on Barrett’s esophagus. IVW: inverse variance weighted method with multiplicative random-effects; WM: weighted median estimator. Notably, the corresponding estimates from MR-Egger regression for *Actinomyces*, *Streptococcus*, and *Veillonella* were not showed herein due to the wide confidence intervals.

Figure 3 shows the associations of genetically predicted esophagus-specific genera of gut microbiota with BE, with complete results presented in Additional file 1: Supplementary Table S4. Genetically predicted higher *Actinomyces* was significantly associated with lower BE risk, with no heterogeneity and horizontal pleiotropy but adequate power. In addition, higher *Veillonella* was suggestively associated with lower BE risk, with no heterogeneity and horizontal pleiotropy. Though sensitivity analyses yield similar results, none of them reached the Bonferroni-corrected significance.

## Discussion

### Principal Findings

This MR study, taking advantage of the largest publicly available GWAS on gut microbiota and BE, was the first extensive analysis investigating the potential roles of a broad range of the dominant genera of human gut microbiota in BE. Our study found that genetically predicted higher *Actinomyces* and higher *Ruminiclostridium* appeared to protect against BE. Our study also found that genetically predicted higher *Alistipes*, higher *Eubacterium*, and higher *Veillonella* were suggestively associated with lower BE risk, while higher *Faecalibacterium* was associated with higher BE risk.

### Comparison With Other Studies

In particular, there was no evidence supporting an association of *Streptococcus* with BE in our study, in which *Streptococcus* was thought to be the dominant BE-specific microbiota (Yang et al., 2009; Gall et al., 2015). This finding is inconsistent with that of previous observational studies showing that higher *Streptococcus* was positively associated with BE (Lopetuso et al., 2020). However, the discrepancies between previous findings and our study may be due to the chance finding caused by the small sample size and the unmeasured confounding caused by other factors, such as obesity and health status.

Our findings were consistent with those of previous studies showing that higher *Actinomyces* and higher *Veillonella* were inversely associated with BE (Elliott et al., 2017; Snider et al., 2019; Zhou et al., 2020), although the *Veillonella*-BE association became less evident after adjusting for multiplicity. However, our findings did not show any association of *Prevotella* with BE risk, which was inconsistent with previous observational studies showing a possible association of *Prevotella* with BE/EAC (Elliott et al., 2017; Snider et al., 2019; Lopetuso et al., 2020). Such discrepancies may be the consequence of confounding bias in observational studies arising from the diet, environmental factors, or medication (Rothschild et al., 2018; Hills et al., 2019), which were thought to shape the composition and abundance of genera of gut microbiota in humans in real-time, probably through mediations of metabolites and inflammatory cytokines (e.g., IL-8) (Munch et al., 2019).

Our findings also suggested that higher *Alistipes* appeared to protect against BE, which was not reported in previous studies. Nevertheless, from a biological perspective, higher *Alistipes* (i.e., a recently discovered gram-negative and anaerobic genus of the *Bacteroidetes* phylum in mostly healthy human GI tract) acts as a potential pathogen, contributing to the beneficial immunomodulation in cancer [e.g., colorectal cancer (Moschen et al., 2016)]. Second, *Alistipes* has been reported to modulate the tumor microenvironment and gut inflammation (Parker et al., 2020), and thus might have a role in cancer immunotherapy. For instance, manipulating the tumor microenvironment by reducing tumor necrosis factor produced myeloid cells using antibiotics appeared to decrease the tumor eradication rate (Iida et al., 2013). Furthermore, patients with non–small-cell lung cancer who responded to nivolumab (a checkpoint inhibitor for PD-1) tended to have an elevated *Alistipes* (Jin et al., 2019). Third, *Alistipes*-related dysbiosis has already been implicated in several other diseases (e.g., liver fibrosis (Rau et al., 2018), cardiovascular diseases (Zuo et al., 2019), and mood disorder (Bangsgaard Bendtsen et al., 2012)), possibly *via* inflammation.

In addition, our findings suggested that higher *Faecalibacterium* [i.e., a gram-positive and anaerobic bacterium that is one of the most abundant and critical commensal bacteria of human gut microbiota in the intestine (Miquel et al., 2013; Bag et al., 2017)] appeared to increase BE, possibly due to its role in boosting the immune system (Miquel et al., 2013) and improving gut barrier function (Stenman et al., 2016). Similar effects were also reported in Crohn’s disease (Wright et al., 2015). Finally, our findings suggested that higher *Eubacterium* (a gram-positive genus of the *Eubacteriaceae* family) and higher *Ruminiclostridium* may also have roles in reducing BE risk. However, further research studies using randomized controlled trials remain required to verify such findings.

### Limitations

Although MR provides less confounded estimates of the gut microbiota–BE associations, limitations still exist. First, MR estimates rest on stringent assumptions (e.g., the independence and exclusion–restriction assumptions), which are always untestable (Glymour et al., 2012). However, in this study, we selected genetic instruments that strongly predicted gut microbiota from the largest publicly available GWAS, with an F statistic being greater than 10 to reduce the possibility of weak instrument bias (Bowden et al., 2016a). Furthermore, we selected valid genetic instruments at a “suggestive” threshold of *p* < 5 × 10^–6^, instead of the traditional genome-wide significance (i.e., *p* < 5 × 10^–8^), which may induce weak instrument bias. However, the sensitivity analyses, particularly MR-RAPS allowing the inclusion of weak genetic instruments (Zhao et al., 2020), yielded consistent results, indicating a less likely weak instrument bias. We excluded instruments associated with any potential confounders of the gut microbiota–BE associations and any known pleiotropic effects to reduce the risk of violating the independence and exclusion–restriction assumptions. We also conducted Cochran’s Q statistic test to detect the potential heterogeneity of the causal estimates and MR-Egger regression to examine the possible pleiotropic effects. No heterogeneity and pleiotropy were noted, as shown in Figures 1–3 and Additional file 1: Supplementary Table S4.

Second, the effects of genetic instruments on gut microbiota composition and abundance vary considerably across GWAS studies, and seldom do replications across these studies be available (Kurilshikov et al., 2017). As such, population-specific microbiota compositions may exist, which may result in a broad uncertainty and a lack of reproduction of our findings. However, the use of the largest publicly available GWAS of MiBioGen and extensive sensitivity analyses may reduce the risk of such issues. Furthermore, genetic instruments typically explain a small variation in a specific genus of gut microbiota, inducing underpowered MR estimates. However, our power analysis shows adequate power (i.e., 80%) to detect a positive association at the α = 0.05 level, given the true association exists.

Third, we limited MR analyses to participants of mainly European ancestry to reduce the risk of population stratification. Thus, our findings may not extend to other populations, although causations are always consistent across populations (Lopez et al., 2019). In addition, summary statistics obtained from GWAS on genera of gut microbiota had partially overlapping sets of participants from UK Biobank, which may bias our estimates with uncertain magnitudes and directions (Burgess et al., 2016). However, such an impact seemed small as MR-RAPS robust to the overlapping sample issue yielded similar results (Zhao et al., 2020).

Fourth, canalization buffering genetic factors may also exist. However, its impact on our estimates remains unknown. Fifth, our causal estimates reflected the natural genetic variation in lifelong exposure of genera of gut microbiota on BE, which might be different from the effects of short-term interventions with antibiotics.

### Public Health Implications

Nevertheless, our findings provide genetic evidence for the associations of several genera of human gut microbiota with the risk of developing BE. Replication using different populations when the large-scale GWAS becomes available remains valuable to cross-validate our findings. Furthermore, manipulation of the abundance of *Actinomyces* and *Ruminiclostridium* may be helpful in preventing BE. However, *Actinomyces* are opportunistic pathogens in humans and may cause the infection of actinomycosis (Kononen and Wade, 2015), which should be considered. In addition, future research involving the associations of gut microbiota and BE might prioritize these four genera of gut microbiota (i.e., *Alistipes*, *Eubacterium*, *Veillonella*, and *Faecalibacterium*).

## Conclusion

Our MR study showed that higher *Actinomyces* and higher *Ruminiclostridium* might have roles in preventing BE. Our study also suggested the potential benefit of higher *Alistipes*, *Eubacterium*, and *Veillonella*, and lower *Faecalibacterium* in preventing BE. However, a better understanding of their etiological roles in BE could provide additional insights and be valuable to reduce the burden of BE and EAC worldwide.

## Data Availability

Publicly available datasets were analyzed in this study. The names of the repository/repositories and accession number(s) can be found in the article/Supplementary Material; further inquiries can be directed to the corresponding author.
