# Supplementary material for: Roles of 21 Genera of Human Gut Microbiota in Barrett’s Esophagus Risk: A Mendelian Randomization Study
Source: Front Genet. 2022 Jun 9;13:894900. doi: 10.3389/fgene.2022.894900 (PMC9219910; doi:10.3389/fgene.2022.894900)
Supplement: Supplementary file 1 [file DataSheet1.docx]

**Additional files**

**Table S1.** Genetic instruments’ associations with 21 genera of gut microbiota and Barrett’s Esophagus used in this study

| **Genus with MiBioGen ID** | **SNP** | **Effect/Other allele** | **Effect allele frequency** | **R^2 a^** | **β_GX_ (SE(β_GX_))** | ***P* for β_GX_** | **β_GY_ (SE(β_GY_))** | ***P* for β_GY_** |
| --- | --- | --- | --- | --- | --- | --- | --- | --- |
| Eubacteriumruminantiumgroup.id.11340 | rs10923018 | G/A | 0.48 | 0.0026 | 0.073(0.016) | 6.80E-06 | -0.020(0.015) | 0.193 |
|  | rs11637981 | G/T | 0.50 | 0.0027 | -0.073(0.016) | 5.40E-06 | -0.013(0.015) | 0.381 |
|  | rs16891896 | G/A | 0.06 | 0.0042 | -0.175(0.039) | 2.40E-06 | 0.021(0.035) | 0.553 |
|  | rs17519472 | C/T | 0.15 | 0.0027 | 0.108(0.023) | 4.70E-06 | -0.019(0.022) | 0.373 |
|  | rs209813 | G/A | 0.16 | 0.0026 | -0.103(0.024) | 9.20E-06 | -0.006(0.023) | 0.812 |
|  | rs2116427 | A/G | 0.25 | 0.0032 | 0.091(0.018) | 4.70E-07 | -0.007(0.017) | 0.679 |
|  | rs2229917 | A/G | 0.06 | 0.0032 | 0.154(0.032) | 2.20E-06 | -0.004(0.031) | 0.889 |
|  | rs2817174 | C/T | 0.42 | 0.0026 | -0.073(0.016) | 7.90E-06 | 0.005(0.016) | 0.756 |
|  | rs606117 | A/G | 0.34 | 0.0029 | 0.083(0.018) | 4.80E-06 | -0.018(0.016) | 0.280 |
|  | rs7000472 | A/G | 0.36 | 0.0027 | -0.076(0.017) | 4.10E-06 | 0.031(0.015) | 0.042 |
| Actinomyces.id.423 | rs10787984 | G/C | 0.17 | 0.0026 | 0.094(0.021) | 9.60E-06 | -0.021(0.020) | 0.302 |
|  | rs2715439 | C/T | 0.44 | 0.0027 | 0.075(0.016) | 6.30E-06 | -0.018(0.015) | 0.234 |
|  | rs4146653 | G/A | 0.18 | 0.0028 | 0.099(0.021) | 4.50E-06 | -0.035(0.020) | 0.074 |
| Alistipes.id.968 | rs1107244 | G/A | 0.08 | 0.0011 | 0.076(0.017) | 3.60E-06 | 0.005(0.027) | 0.859 |
|  | rs11958296 | A/G | 0.07 | 0.0012 | -0.098(0.022) | 9.30E-06 | 0.045(0.029) | 0.125 |
|  | rs1689282 | A/C | 0.35 | 0.0012 | -0.052(0.011) | 5.30E-06 | 0.007(0.016) | 0.674 |
|  | rs2290844 | C/T | 0.11 | 0.0010 | 0.081(0.019) | 9.10E-06 | 0.030(0.028) | 0.280 |
|  | rs2875322 | T/C | 0.21 | 0.0011 | -0.058(0.013) | 8.80E-06 | 0.029(0.019) | 0.117 |
|  | rs4810359 | A/G | 0.14 | 0.0011 | -0.065(0.015) | 7.50E-06 | 0.029(0.021) | 0.161 |
|  | rs7129639 | C/A | 0.39 | 0.0013 | -0.052(0.011) | 1.80E-06 | 0.037(0.016) | 0.017 |
|  | rs8130320 | A/G | 0.49 | 0.0012 | -0.049(0.011) | 4.80E-06 | 0.001(0.015) | 0.969 |
| Bacteroides.id.918 | rs17619981 | T/G | 0.08 | 0.0014 | 0.088(0.019) | 2.70E-06 | 0.027(0.025) | 0.280 |
|  | rs2366421 | T/A | 0.23 | 0.0011 | -0.053(0.012) | 7.60E-06 | 0.010(0.017) | 0.540 |
|  | rs495004 | C/G | 0.23 | 0.0012 | -0.061(0.013) | 3.40E-06 | -0.014(0.019) | 0.457 |
|  | rs6795673 | C/T | 0.43 | 0.0014 | 0.054(0.011) | 3.40E-07 | 0.010(0.015) | 0.503 |
| Bifidobacterium.id.436 | rs10841473 | G/C | 0.26 | 0.0016 | -0.062(0.013) | 1.60E-06 | -0.012(0.017) | 0.478 |
|  | rs12022129 | G/A | 0.22 | 0.0013 | 0.062(0.014) | 8.00E-06 | 0.021(0.019) | 0.259 |
|  | rs1961273 | C/T | 0.27 | 0.0018 | 0.067(0.013) | 3.50E-07 | 0.005(0.018) | 0.799 |
|  | rs5746486 | T/C | 0.36 | 0.0013 | -0.054(0.012) | 9.00E-06 | -0.009(0.016) | 0.567 |
|  | rs857444 | C/T | 0.35 | 0.0014 | 0.056(0.012) | 3.60E-06 | -0.008(0.016) | 0.625 |
| Blautia.id.1992 | rs11149971 | C/T | 0.06 | 0.0016 | 0.118(0.023) | 1.00E-06 | -0.026(0.033) | 0.430 |
|  | rs16892041 | T/C | 0.19 | 0.0011 | -0.062(0.014) | 8.80E-06 | -0.010(0.021) | 0.625 |
|  | rs2788271 | T/G | 0.17 | 0.0010 | -0.058(0.013) | 7.20E-06 | -0.024(0.020) | 0.228 |
|  | rs3005511 | A/G | 0.33 | 0.0011 | 0.050(0.011) | 6.20E-06 | 0.008(0.016) | 0.620 |
|  | rs3005511 | A/G | 0.33 | 0.0011 | 0.050(0.011) | 6.20E-06 | 0.008(0.016) | 0.620 |
|  | rs4926264 | T/C | 0.09 | 0.0012 | 0.083(0.018) | 5.10E-06 | -0.033(0.029) | 0.254 |
|  | rs682885 | A/G | 0.41 | 0.0012 | -0.049(0.011) | 4.50E-06 | -0.010(0.015) | 0.504 |
| Butyricicoccus.id.2055 | rs12585793 | T/C | 0.06 | 0.0068 | -0.262(0.056) | 5.80E-06 | -0.042(0.038) | 0.270 |
|  | rs7322368 | T/C | 0.10 | 0.0012 | 0.082(0.018) | 5.50E-06 | -0.016(0.026) | 0.535 |
| Dorea.id.1997 | rs12216169 | T/A | 0.09 | 0.0013 | 0.088(0.019) | 5.30E-06 | 0.011(0.028) | 0.685 |
|  | rs12537781 | T/C | 0.23 | 0.0011 | -0.056(0.013) | 9.20E-06 | 0.017(0.017) | 0.316 |
|  | rs1899291 | C/T | 0.12 | 0.0012 | 0.070(0.015) | 4.60E-06 | 0.012(0.022) | 0.603 |
|  | rs3005511 | A/G | 0.33 | 0.0012 | 0.052(0.011) | 5.30E-06 | 0.008(0.016) | 0.620 |
|  | rs3005511 | A/G | 0.33 | 0.0012 | 0.052(0.011) | 5.30E-06 | 0.008(0.016) | 0.620 |
|  | rs3752849 | G/A | 0.03 | 0.0037 | 0.164(0.037) | 7.70E-06 | -0.051(0.040) | 0.199 |
|  | rs4793307 | C/T | 0.24 | 0.0012 | 0.057(0.012) | 4.00E-06 | 0.028(0.017) | 0.100 |
| Faecalibacterium.id.2057 | rs11776390 | T/C | 0.09 | 0.0012 | -0.078(0.017) | 6.40E-06 | -0.020(0.025) | 0.411 |
|  | rs12320842 | C/G | 0.13 | 0.0019 | 0.095(0.016) | 7.60E-09 | 0.015(0.024) | 0.527 |
|  | rs1271565 | C/T | 0.28 | 0.0013 | -0.058(0.012) | 1.30E-06 | -0.007(0.017) | 0.670 |
|  | rs12753492 | A/C | 0.15 | 0.0010 | 0.064(0.015) | 8.80E-06 | -0.011(0.022) | 0.619 |
|  | rs2835874 | T/C | 0.06 | 0.0011 | -0.087(0.020) | 7.50E-06 | -0.025(0.029) | 0.395 |
| Fusicatenibacter.id.11305 | rs1864685 | A/C | 0.47 | 0.0012 | -0.049(0.011) | 5.00E-06 | -0.008(0.015) | 0.611 |
|  | rs2039204 | T/A | 0.42 | 0.0012 | -0.050(0.011) | 3.90E-06 | -0.009(0.015) | 0.530 |
|  | rs206581 | A/G | 0.19 | 0.0011 | -0.057(0.013) | 9.00E-06 | 0.022(0.018) | 0.220 |
|  | rs4378146 | A/C | 0.23 | 0.0014 | -0.062(0.013) | 7.20E-07 | -0.018(0.017) | 0.309 |
|  | rs6515626 | G/A | 0.06 | 0.0026 | 0.142(0.031) | 7.30E-06 | -0.001(0.037) | 0.985 |
|  | rs792108 | T/C | 0.37 | 0.0012 | -0.051(0.011) | 8.50E-06 | -0.000(0.016) | 0.985 |
|  | rs8028026 | A/G | 0.10 | 0.0012 | -0.079(0.018) | 8.10E-06 | 0.039(0.024) | 0.101 |
|  | rs9905659 | G/A | 0.18 | 0.0012 | -0.062(0.014) | 7.30E-06 | -0.011(0.020) | 0.574 |
| Haemophilus.id.3698 | rs12191680 | C/G | 0.17 | 0.0031 | 0.107(0.020) | 1.50E-07 | 0.004(0.021) | 0.847 |
|  | rs12876183 | T/A | 0.28 | 0.0022 | 0.075(0.017) | 9.60E-06 | -0.007(0.017) | 0.697 |
|  | rs4822728 | T/C | 0.44 | 0.0024 | 0.071(0.015) | 3.50E-06 | 0.047(0.016) | 0.003 |
|  | rs9328464 | T/C | 0.48 | 0.0026 | 0.072(0.015) | 1.40E-06 | 0.041(0.015) | 0.007 |
|  | rs9574096 | A/T | 0.35 | 0.0025 | -0.074(0.016) | 2.20E-06 | 0.002(0.016) | 0.898 |
|  | rs9895850 | T/C | 0.04 | 0.0046 | -0.193(0.042) | 2.10E-06 | -0.008(0.035) | 0.817 |
| Lachnoclostridium.id.11308 | rs1031599 | G/T | 0.08 | 0.0012 | -0.079(0.018) | 6.30E-06 | -0.023(0.028) | 0.419 |
|  | rs1997204 | T/C | 0.05 | 0.0014 | -0.108(0.024) | 6.00E-06 | 0.009(0.033) | 0.795 |
|  | rs4738679 | G/A | 0.34 | 0.0012 | -0.052(0.011) | 4.40E-06 | 0.062(0.016) | 0.000 |
|  | rs789029 | C/T | 0.19 | 0.0012 | -0.064(0.014) | 3.80E-06 | -0.003(0.020) | 0.885 |
| Lactobacillus.id.1837 | rs12693845 | C/T | 0.36 | 0.0030 | -0.081(0.018) | 9.00E-06 | 0.036(0.016) | 0.024 |
|  | rs16861661 | G/A | 0.05 | 0.0043 | -0.183(0.038) | 1.30E-06 | -0.024(0.031) | 0.449 |
|  | rs328312 | T/A | 0.46 | 0.0033 | 0.082(0.017) | 1.40E-06 | -0.030(0.015) | 0.050 |
|  | rs768253 | T/G | 0.40 | 0.0030 | -0.079(0.017) | 4.20E-06 | -0.009(0.015) | 0.552 |
| Peptococcus.id.2037 | rs11001941 | G/A | 0.06 | 0.0044 | -0.196(0.039) | 1.30E-06 | -0.029(0.031) | 0.360 |
|  | rs11030569 | A/T | 0.08 | 0.0040 | -0.174(0.037) | 3.10E-06 | 0.009(0.031) | 0.762 |
| Peptococcus.id.2037 | rs12069354 | C/T | 0.07 | 0.0036 | 0.168(0.038) | 9.30E-06 | 0.034(0.031) | 0.267 |
|  | rs6918730 | G/A | 0.10 | 0.0038 | 0.135(0.029) | 1.10E-06 | 0.012(0.024) | 0.607 |
|  | rs7033353 | T/G | 0.44 | 0.0040 | 0.090(0.019) | 2.20E-06 | -0.007(0.015) | 0.630 |
|  | rs7766680 | G/C | 0.24 | 0.0037 | 0.098(0.021) | 3.50E-06 | 0.003(0.017) | 0.873 |
| Prevotella9.id.11183 | rs10512344 | C/G | 0.03 | 0.0067 | 0.247(0.054) | 3.20E-06 | 0.076(0.039) | 0.052 |
|  | rs11199734 | A/T | 0.21 | 0.0020 | 0.077(0.017) | 7.00E-06 | -0.001(0.019) | 0.954 |
|  | rs16966465 | G/C | 0.22 | 0.0020 | 0.074(0.017) | 9.30E-06 | 0.007(0.019) | 0.725 |
|  | rs2104588 | T/C | 0.12 | 0.0021 | 0.106(0.024) | 8.10E-06 | 0.038(0.025) | 0.125 |
|  | rs2495052 | A/G | 0.17 | 0.0020 | 0.084(0.019) | 9.00E-06 | -0.026(0.020) | 0.200 |
|  | rs2683313 | A/G | 0.31 | 0.0022 | -0.072(0.015) | 1.70E-06 | 0.001(0.016) | 0.938 |
|  | rs4968431 | G/T | 0.36 | 0.0019 | 0.064(0.014) | 8.60E-06 | 0.007(0.016) | 0.687 |
|  | rs746764 | T/C | 0.17 | 0.0024 | -0.092(0.019) | 2.00E-06 | 0.040(0.020) | 0.045 |
|  | rs9428102 | A/G | 0.24 | 0.0019 | -0.078(0.018) | 4.60E-06 | 0.018(0.018) | 0.324 |
|  | rs9613013 | G/A | 0.15 | 0.0021 | 0.092(0.020) | 6.10E-06 | 0.049(0.022) | 0.027 |
| Roseburia.id.2012 | rs16910295 | T/C | 0.06 | 0.0013 | -0.098(0.021) | 2.90E-06 | -0.013(0.030) | 0.658 |
|  | rs2034589 | G/C | 0.23 | 0.0015 | 0.063(0.012) | 5.00E-07 | 0.016(0.019) | 0.400 |
|  | rs2160994 | T/C | 0.38 | 0.0014 | 0.055(0.011) | 9.70E-07 | -0.009(0.016) | 0.559 |
|  | rs2943022 | T/C | 0.47 | 0.0012 | 0.049(0.011) | 4.10E-06 | -0.013(0.015) | 0.378 |
|  | rs4748237 | G/C | 0.41 | 0.0012 | 0.049(0.011) | 4.70E-06 | 0.031(0.015) | 0.043 |
|  | rs6445851 | G/A | 0.40 | 0.0012 | -0.050(0.011) | 3.50E-06 | -0.012(0.015) | 0.441 |
|  | rs6930661 | C/T | 0.06 | 0.0013 | -0.096(0.020) | 2.50E-06 | -0.006(0.029) | 0.847 |
| Ruminiclostridium5.id.11355 | rs1492620 | T/C | 0.08 | 0.0012 | -0.083(0.018) | 3.50E-06 | 0.055(0.026) | 0.034 |
|  | rs2286384 | G/C | 0.51 | 0.0013 | -0.052(0.011) | 1.40E-06 | 0.019(0.015) | 0.213 |
|  | rs2791343 | T/C | 0.33 | 0.0012 | 0.052(0.011) | 5.50E-06 | -0.006(0.016) | 0.716 |
|  | rs2801960 | C/G | 0.32 | 0.0012 | 0.052(0.012) | 6.20E-06 | -0.024(0.016) | 0.143 |
|  | rs2833828 | G/A | 0.46 | 0.0012 | 0.049(0.011) | 6.80E-06 | -0.001(0.015) | 0.946 |
|  | rs4955951 | A/G | 0.09 | 0.0011 | -0.071(0.017) | 1.00E-05 | 0.021(0.024) | 0.373 |
|  | rs6121460 | G/A | 0.08 | 0.0013 | 0.093(0.020) | 2.60E-06 | 0.010(0.030) | 0.749 |
|  | rs8053158 | A/G | 0.11 | 0.0012 | -0.074(0.016) | 5.90E-06 | 0.032(0.023) | 0.169 |
| Ruminococcus1.id.11373 | rs10995816 | C/G | 0.10 | 0.0011 | -0.076(0.017) | 8.40E-06 | 0.021(0.025) | 0.400 |
|  | rs4849717 | T/A | 0.06 | 0.0019 | 0.133(0.030) | 8.80E-06 | -0.001(0.036) | 0.982 |
|  | rs7117576 | A/G | 0.09 | 0.0014 | 0.083(0.017) | 6.50E-07 | 0.034(0.024) | 0.148 |
|  | rs7583465 | C/T | 0.38 | 0.0013 | 0.053(0.011) | 2.60E-06 | -0.009(0.015) | 0.546 |
| Streptococcus.id.1853 | rs10028567 | C/T | 0.09 | 0.0014 | -0.092(0.019) | 7.30E-06 | -0.028(0.026) | 0.290 |
|  | rs6563952 | G/C | 0.09 | 0.0013 | 0.083(0.018) | 5.80E-06 | 0.011(0.026) | 0.677 |
|  | rs6806351 | T/C | 0.21 | 0.0013 | -0.063(0.014) | 4.90E-06 | 0.034(0.018) | 0.056 |
| Subdoligranulum.id.2070 | rs10065321 | T/C | 0.38 | 0.0013 | -0.051(0.011) | 2.10E-06 | 0.015(0.016) | 0.334 |
|  | rs10497836 | C/T | 0.27 | 0.0011 | -0.052(0.012) | 8.40E-06 | -0.028(0.017) | 0.103 |
|  | rs12638227 | G/C | 0.47 | 0.0015 | -0.056(0.011) | 2.50E-07 | -0.001(0.015) | 0.944 |
|  | rs16962433 | A/T | 0.10 | 0.0013 | 0.086(0.019) | 7.60E-06 | 0.027(0.026) | 0.289 |
|  | rs4347804 | A/G | 0.04 | 0.0050 | 0.166(0.036) | 2.20E-06 | -0.024(0.038) | 0.519 |
|  | rs6555306 | T/C | 0.12 | 0.0013 | -0.074(0.016) | 2.80E-06 | -0.000(0.023) | 0.991 |
| Veillonella.id.2198 | rs11614532 | G/C | 0.30 | 0.0024 | 0.074(0.017) | 7.10E-06 | -0.030(0.016) | 0.065 |
|  | rs1882878 | A/G | 0.29 | 0.0024 | -0.077(0.016) | 3.00E-06 | 0.035(0.017) | 0.042 |
|  | rs2013594 | T/C | 0.36 | 0.0023 | -0.072(0.016) | 3.40E-06 | -0.002(0.016) | 0.894 |

**^a^** R^2^: the variance of each genus of gut microbiota explained by the included genetic instruments.

**Table S2.** Study details for these well-acknowledged confounders of the gut microbiota-Barrett’s esophagus association

| **Potential confounders** | **N, # of cases/# of controls** | **Age (years)** | **Male sex (%)** | **Summary statistics,**  **mean (SD)** | **Year** | **PMID** |
| --- | --- | --- | --- | --- | --- | --- |
| Waist hip ratio | 697,734 | 18-107 | 315,284 (45.4) | 0.87 (0.09) | 2019 | 30239722 |
| Cigarettes per day (ieu-b-25) | 337,334 | N.A. | N.A. | 2.35 (0.97 cigarettes) **^b^** | 2019 | 30643251 |
| Drinks per week (ieu-b-73) | 941,280 | N.A. | N.A. | 7.84 (2.88 drinks) **^c^** | 2019 | 30643251 |
| Depression **^a^** | 246,363/561,190 | N.A. | ~326,029 (~40.3) | N.A. | 2019 | 30718901 |
| Years of schooling (ieu-a-1239) | Up to 1,131,881 | ~63.4 | ~531,718 (~57.0) | 13.34 (4.2 years) | 2018 | 30038396 |

**Note:**

^a^ Depression cases are defined by the participants’ response to the questions “Have you ever seen a general practitioner for nerves, anxiety, tension or depression?” or “Have you ever seen a psychiatrist for nerves, anxiety, tension or depression?” or responses to web-based surveys, with individuals that self-reported as having received a clinical diagnosis or treatment for depression.

^b^ The weighted mean and standard deviation of cigarettes per day and drinks per week, with the weights being the proportion of each sub-cohort.

^c^ The weighted age of initial smoking, with the weights being the proportion of sample size for each sub-cohort.

**Table S3.** The identified pleiotropic effects of genetic instruments excluded from this study

| **Genus** | **SNP** | **Trait** | **Ancestry** | **Year** | **β** | **SE(β)** | ***P*** | **Dataset** |
| --- | --- | --- | --- | --- | --- | --- | --- | --- |
| Bifidobacterium.id.436 | rs182549 | 15 anhydro glucitol levels | Mixed | 2017 | 16.91 | 1 | 4.00E-64 | NHGRI-EBI_GWAS_Catalog |
|  | rs182549 | Parkinson’s disease | European | 2014 | -0.1054 | 0.01996 | 9.41E-09 | Nalls-M_Parkinsons-Disease_EUR_2014 |
|  | rs182549 | Arm fat mass left | European | 2017 | -0.01634 | 0.002822 | 7.09E-09 | Neale-B_UKBB_EUR_2017 |
|  | rs182549 | Arm fat mass right | European | 2017 | -0.01649 | 0.002822 | 5.13E-09 | Neale-B_UKBB_EUR_2017 |
|  | rs182549 | Arm fat percentage left | European | 2017 | -0.01236 | 0.00218 | 1.41E-08 | Neale-B_UKBB_EUR_2017 |
|  | rs182549 | Arm fat percentage right | European | 2017 | -0.01263 | 0.00219 | 8.09E-09 | Neale-B_UKBB_EUR_2017 |
|  | rs182549 | Body fat percentage | European | 2017 | -0.01385 | 0.002208 | 3.51E-10 | Neale-B_UKBB_EUR_2017 |
|  | rs182549 | Body mass index | European | 2017 | -0.01633 | 0.002849 | 9.97E-09 | Neale-B_UKBB_EUR_2017 |
|  | rs182549 | Forced vital capacity | European | 2017 | 0.01433 | 0.002337 | 8.81E-10 | Neale-B_UKBB_EUR_2017 |
|  | rs182549 | Leg fat mass left | European | 2017 | -0.01403 | 0.002276 | 7.05E-10 | Neale-B_UKBB_EUR_2017 |
|  | rs182549 | Leg fat mass right | European | 2017 | -0.01391 | 0.002302 | 1.54E-09 | Neale-B_UKBB_EUR_2017 |
|  | rs182549 | Leg fat percentage left | European | 2017 | -0.01057 | 0.001801 | 4.29E-09 | Neale-B_UKBB_EUR_2017 |
|  | rs182549 | Leg fat percentage right | European | 2017 | -0.01094 | 0.001824 | 1.97E-09 | Neale-B_UKBB_EUR_2017 |
|  | rs182549 | Trunk fat mass | European | 2017 | -0.01721 | 0.002899 | 2.95E-09 | Neale-B_UKBB_EUR_2017 |
|  | rs182549 | Trunk fat percentage | European | 2017 | -0.01606 | 0.002647 | 1.30E-09 | Neale-B_UKBB_EUR_2017 |
|  | rs182549 | Whole body fat mass | European | 2017 | -0.01655 | 0.00281 | 3.85E-09 | Neale-B_UKBB_EUR_2017 |
| Streptococcus.id.1853 | rs17708276 | Irritability | European | 2017 | 0.00916 | 0.00163 | 1.93E-08 | Neale-B_UKBB_EUR_2017 |
|  | rs17708276 | Leg fat-free mass left | European | 2017 | -0.01274 | 0.002336 | 4.98E-08 | Neale-B_UKBB_EUR_2017 |
|  | rs17708276 | Neuroticism score | European | 2017 | 0.0953 | 0.0127 | 6.31E-14 | Neale-B_UKBB_EUR_2017 |
|  | rs17708276 | Vascular or heart problems diagnosed by doctor: high blood pressure | European | 2017 | -0.009191 | 0.001578 | 5.74E-09 | Neale-B_UKBB_EUR_2017 |
|  | rs17708276 | Worrier or anxious feelings | European | 2017 | 0.01269 | 0.001761 | 5.72E-13 | Neale-B_UKBB_EUR_2017 |
|  | rs17708276 | Worry too long after embarrassment | European | 2017 | 0.01062 | 0.001795 | 3.32E-09 | Neale-B_UKBB_EUR_2017 |

**Table S4**. Associations of genetically predicted genera of gut microbiota on Barrett’s esophagus using Mendelian randomization, including inverse variance weighted method (IVW), weighted median (WM), MR Egger, MR robust adjusted profile score (MR-RAPS), and MR-PRESSO

| **Site** | **Genus** | **Method** | **# SNPs** | **Odds Ratio**  **(95% CI)** | ***P*** | **MR Egger Intercept (SE)** | ***P* for MR Egger Intercept** | ***P* for Cochran’s Q** | **Power** |
| --- | --- | --- | --- | --- | --- | --- | --- | --- | --- |
| Gut (multiple ancestry) | *Alistipes* | IVW | 8 | 0.77 (0.61 to 0.99) | 0.040 |  |  | 0.279 | 1.00 |
|  |  | WM | 8 | 0.74 (0.56 to 0.99) | 0.045 |  |  |  | 1.00 |
|  |  | MR Egger | 8 | 0.99 (0.30 to 3.33) | 0.990 | -0.015 (0.038) | 0.6956 |  | 0.05 |
|  |  | MR-RAPS | 8 | 0.75 (0.58 to 0.97) | 0.027 |  |  |  | 1.00 |
|  |  | MR PRESSO | 8 | 0.77 (0.61 to 0.99) | 0.080 |  |  |  | 1.00 |
|  | *Bacteroides* | IVW | 4 | 1.16 (0.94 to 1.43) | 0.170 |  |  | 0.668 | 0.79 |
|  |  | WM | 4 | 1.24 (0.86 to 1.79) | 0.251 |  |  |  | 0.98 |
|  |  | MR Egger | 4 | 2.21 (0.47 to 10.48) | 0.423 | -0.040 (0.048) | 0.4951 |  | 1.00 |
|  |  | MR-RAPS | 4 | 1.16 (0.85 to 1.59) | 0.340 |  |  |  | 0.81 |
|  |  | MR PRESSO | 4 | 1.16 (0.94 to 1.43) | 0.264 |  |  |  | 0.79 |
|  | *Blautia* | IVW | 6 | 1.04 (0.83 to 1.32) | 0.716 |  |  | 0.521 | 0.17 |
|  |  | WM | 6 | 1.17 (0.84 to 1.64) | 0.356 |  |  |  | 0.96 |
|  |  | MR Egger | 6 | 0.51 (0.20 to 1.27) | 0.220 | 0.047 (0.029) | 0.1823 |  | 1.00 |
|  |  | MR-RAPS | 6 | 1.05 (0.80 to 1.38) | 0.747 |  |  |  | 0.18 |
|  |  | MR PRESSO | 6 | 1.04 (0.83 to 1.32) | 0.731 |  |  |  | 0.17 |
|  | *Dorea* | IVW | 6 | 1.02 (0.79 to 1.32) | 0.867 |  |  | 0.305 | 0.09 |
|  |  | WM | 6 | 1.14 (0.83 to 1.57) | 0.415 |  |  |  | 0.95 |
|  |  | MR Egger | 6 | 0.67 (0.32 to 1.37) | 0.332 | 0.031 (0.025) | 0.2833 |  | 1.00 |
|  |  | MR-RAPS | 6 | 1.01 (0.77 to 1.32) | 0.967 |  |  |  | 0.05 |
|  |  | MR PRESSO | 6 | 1.02 (0.79 to 1.32) | 0.874 |  |  |  | 0.09 |
|  | *Faecalibacterium* | IVW | 5 | 1.15 (0.99 to 1.33) | 0.059 |  |  | 0.882 | 0.84 |
|  |  | WM | 5 | 1.17 (0.85 to 1.62) | 0.334 |  |  |  | 0.92 |
|  |  | MR Egger | 5 | 1.67 (0.42 to 6.60) | 0.516 | -0.028 (0.051) | 0.6228 |  | 1.00 |
|  |  | MR-RAPS | 5 | 1.15 (0.87 to 1.52) | 0.321 |  |  |  | 0.84 |
|  |  | MR PRESSO | 5 | 1.15 (0.99 to 1.33) | 0.132 |  |  |  | 0.83 |
|  | *Lachnoclostridium* | IVW | 4 | 0.76 (0.40 to 1.45) | 0.408 |  |  | 0.005 | 0.99 |
|  |  | WM | 4 | 0.99 (0.65 to 1.51) | 0.963 |  |  |  | 0.05 |
|  |  | MR Egger | 4 | 3.53 (0.42 to 29.77) | 0.366 | -0.107 (0.074) | 0.2824 |  | 1.00 |
|  |  | MR-RAPS | 4 | 0.90 (0.52 to 1.54) | 0.693 |  |  |  | 0.46 |
|  |  | MR PRESSO | 4 | 0.76 (0.40 to 1.45) | 0.469 |  |  |  | 0.99 |
|  |  | MR PRESSO (Outlier-correct) ^a^ | 3 | 1.07 (0.87 to 1.31) | 0.592 |  |  |  | 0.22 |
|  | *Roseburia* | IVW | 7 | 1.13 (0.91 to 1.40) | 0.288 |  |  | 0.46 | 0.82 |
|  |  | WM | 7 | 1.13 (0.83 to 1.55) | 0.433 |  |  |  | 0.86 |
|  |  | MR Egger | 7 | 1.04 (0.39 to 2.75) | 0.940 | 0.005 (0.029) | 0.8768 |  | 0.15 |
|  |  | MR RAPS | 7 | 1.12 (0.87 to 1.45) | 0.382 |  |  |  | 0.79 |
|  |  | MR PRESSO | 7 | 1.13 (0.91 to 1.40) | 0.329 |  |  |  | 0.82 |
|  | *Ruminococcus* | IVW | 4 | 1.01 (0.75 to 1.35) | 0.953 |  |  | 0.368 | 0.05 |
|  |  | WM | 4 | 0.94 (0.66 to 1.32) | 0.707 |  |  |  | 0.23 |
|  |  | MR Egger | 4 | 1.31 (0.44 to 3.86) | 0.675 | -0.020 (0.041) | 0.6694 |  | 1.00 |
|  |  | MR-RAPS | 4 | 1.00 (0.74 to 1.36) | 0.991 |  |  |  | 0.05 |
|  |  | MR PRESSO | 4 | 1.01 (0.75 to 1.35) | 0.957 |  |  |  | 0.05 |
|  | *Subdoligranulum* | IVW | 6 | 1.04 (0.83 to 1.31) | 0.741 |  |  | 0.413 | 0.18 |
|  |  | WM | 6 | 1.01 (0.74 to 1.36) | 0.971 |  |  |  | 0.05 |
|  |  | MR Egger | 6 | 0.88 (0.44 to 1.76) | 0.741 | 0.012 (0.024) | 0.645 |  | 0.90 |
|  |  | MR-RAPS | 6 | 1.03 (0.81 to 1.33) | 0.792 |  |  |  | 0.15 |
|  |  | MR PRESSO | 6 | 1.04 (0.83 to 1.31) | 0.754 |  |  |  | 0.18 |
| Gut (European ancestry) | *Butyricicoccus* | IVW | 2 | 1.10 (0.84 to 1.44) | 0.473 |  |  | 0.304 | 0.61 |
|  |  | MR-RAPS | 2 | 1.10 (0.84 to 1.46) | 0.486 |  |  |  | 0.62 |
|  | *Eubacterium* | IVW | 10 | 0.89 (0.80 to 0.99) | 0.025 |  |  | 0.747 | 1.00 |
|  |  | WM | 10 | 0.91 (0.77 to 1.07) | 0.244 |  |  |  | 0.98 |
|  |  | MR Egger | 10 | 0.98 (0.60 to 1.62) | 0.951 | -0.009 (0.023) | 0.6911 |  | 0.11 |
|  |  | MR-RAPS | 10 | 0.89 (0.77 to 1.01) | 0.079 |  |  |  | 1.00 |
|  |  | MR PRESSO | 10 | 0.89 (0.80 to 0.99) | 0.051 |  |  |  | 1.00 |
|  | *Fusicatenibacter* | IVW | 8 | 1.00 (0.82 to 1.21) | 0.986 |  |  | 0.516 | 0.05 |
|  |  | WM | 8 | 1.08 (0.83 to 1.41) | 0.551 |  |  |  | 0.58 |
|  |  | MR Egger | 8 | 0.77 (0.36 to 1.65) | 0.524 | 0.017 (0.024) | 0.5106 |  | 1.00 |
|  |  | MR-RAPS | 8 | 1.01 (0.81 to 1.26) | 0.924 |  |  |  | 0.06 |
|  |  | MR PRESSO | 8 | 1.00 (0.82 to 1.21) | 0.986 |  |  |  | 0.05 |
|  | *Ruminiclostridium* | IVW | 8 | 0.75 (0.63 to 0.90) | 0.001 |  |  | 0.714 | 1.00 |
|  |  | WM | 8 | 0.72 (0.54 to 0.97) | 0.029 |  |  |  | 1.00 |
|  |  | MR Egger | 8 | 0.66 (0.24 to 1.78) | 0.442 | 0.008 (0.031) | 0.7955 |  | 1.00 |
|  |  | MR-RAPS | 8 | 0.75 (0.59 to 0.94) | 0.015 |  |  |  | 1.00 |
|  |  | MR PRESSO | 8 | 0.75 (0.63 to 0.90) | 0.016 |  |  |  | 1.00 |
| Esophagus (Multiple ancestry) | *Actinomyces* | IVW | 3 | 0.76 (0.70 to 0.83) | 1.54E-10 |  |  | 0.876 | 1.00 |
|  |  | WM | 3 | 0.78 (0.58 to 1.05) | 0.102 |  |  |  | 1.00 |
|  |  | MR Egger | 3 | 0.59 (0.09 to 3.75) | 0.677 | 0.022 (0.082) | 0.8347 |  | 1.00 |
|  |  | MR-RAPS | 3 | 0.76 (0.59 to 0.97) | 0.030 |  |  |  | 1.00 |
|  | *Bifidobacterium* | IVW | 5 | 1.13 (0.97 to 1.31) | 0.121 |  |  | 0.826 | 0.76 |
|  |  | WM | 5 | 1.17 (0.85 to 1.60) | 0.331 |  |  |  | 0.94 |
|  |  | MR Egger | 5 | 1.80 (0.09 to 35.29) | 0.724 | -0.028 (0.091) | 0.7771 |  | 1.00 |
|  |  | MR-RAPS | 5 | 1.13 (0.87 to 1.47) | 0.357 |  |  |  | 0.78 |
|  |  | MR PRESSO | 5 | 1.13 (0.97 to 1.31) | 0.196 |  |  |  | 0.76 |
|  | *Haemophilus* | IVW | 6 | 1.21 (0.94 to 1.55) | 0.142 |  |  | 0.042 | 1.00 |
|  |  | WM | 6 | 1.04 (0.82 to 1.32) | 0.743 |  |  |  | 0.26 |
|  |  | MR Egger | 6 | 0.81 (0.35 to 1.86) | 0.646 | 0.036 (0.036) | 0.3795 |  | 1.00 |
|  |  | MR-RAPS | 6 | 1.16 (0.90 to 1.50) | 0.238 |  |  |  | 1.00 |
|  |  | MR PRESSO | 6 | 1.21 (0.94 to 1.55) | 0.202 |  |  |  | 1.00 |
|  | *Lactobacillus* | IVW | 4 | 0.88 (0.65 to 1.19) | 0.412 |  |  | 0.045 | 0.94 |
|  |  | WM | 4 | 1.00 (0.80 to 1.27) | 0.972 |  |  |  | 0.05 |
|  |  | MR Egger | 4 | 1.48 (0.54 to 4.03) | 0.525 | -0.050 (0.047) | 0.4013 |  | 1.00 |
|  |  | MR-RAPS | 4 | 0.93 (0.69 to 1.25) | 0.630 |  |  |  | 0.54 |
|  |  | MR PRESSO | 4 | 0.88 (0.65 to 1.19) | 0.472 |  |  |  | 0.94 |
|  | *Peptococcus* | IVW | 6 | 1.06 (0.97 to 1.16) | 0.226 |  |  | 0.838 | 0.57 |
|  |  | WM | 6 | 1.06 (0.89 to 1.27) | 0.518 |  |  |  | 0.62 |
|  |  | MR Egger | 6 | 1.29 (0.81 to 2.08) | 0.346 | -0.027 (0.030) | 0.4299 |  | 1.00 |
|  |  | MR-RAPS | 6 | 1.06 (0.91 to 1.22) | 0.449 |  |  |  | 0.59 |
|  |  | MR PRESSO | 6 | 1.06 (0.97 to 1.16) | 0.280 |  |  |  | 0.57 |
|  | *Prevotella* | IVW | 10 | 1.06 (0.87 to 1.28) | 0.556 |  |  | 0.044 | 0.65 |
|  |  | WM | 10 | 1.06 (0.86 to 1.32) | 0.589 |  |  |  | 0.67 |
|  |  | MR Egger | 10 | 1.55 (0.88 to 2.74) | 0.166 | -0.036 (0.026) | 0.1994 |  | 1.00 |
|  |  | MR-RAPS | 10 | 1.12 (0.91 to 1.38) | 0.276 |  |  |  | 1.00 |
|  |  | MR PRESSO | 10 | 1.06 (0.87 to 1.28) | 0.571 |  |  |  | 0.64 |
|  | *Streptococcus* | IVW | 3 | 0.95 (0.57 to 1.60) | 0.850 |  |  | 0.089 | 0.12 |
|  |  | WM | 3 | 1.15 (0.71 to 1.86) | 0.568 |  |  |  | 0.63 |
|  |  | MR Egger | 3 | 9.08 (1.18 to 69.75) | 0.280 | -0.174 (0.079) | 0.2717 |  | 1.00 |
|  |  | MR-RAPS | 3 | 1.02 (0.62 to 1.67) | 0.950 |  |  |  | 0.06 |
|  | *Veillonella* | IVW | 3 | 0.76 (0.56 to 1.02) | 0.067 |  |  | 0.244 | 1.00 |
|  |  | WM | 3 | 0.69 (0.49 to 0.97) | 0.031 |  |  |  | 1.00 |
|  |  | MR Egger | 3 | 0.00 (0.00 to 6.14) | 0.358 | 0.556 (0.363) | 0.3683 |  | 1.00 |
|  |  | MR-RAPS | 3 | 0.75 (0.56 to 1.00) | 0.052 |  |  |  | 1.00 |

**^a^** Remove the genetic instruments of rs4738629 that was identified as an outlier using MR-PRESSO.
